# When Is a Reservoir Not a Reservoir?

**DOI:** 10.3201/eid0911.030088

**Published:** 2003-11

**Authors:** R.W. Ashford

**Affiliations:** * Liverpool School of Tropical Medicine, Liverpool, United Kingdom

**To the Editor**: Some 80% of parasitic infections of humans are zoonoses (*1*). These infections are caused bymultihost parasites for which the reservoir of infection depends on hosts other than *Homo sapiens*. But what is a reservoir of infection?

Haydon et al. (*2*) proposed a new series of definitions in connection with multihost pathogens, in which a target host is the host of interest in a particular context. The reservoir of infection included, for these authors, all hosts, whether incidental or not, that are epidemiologically connected to (i.e., contribute to transmission to) the target host.

The availability of three terms—reservoir, reservoir of infection, and reservoir host—frequently used interchangeably, leads to confusion. This confusion is, in part, what prompted me (*3*) to slightly redefine a reservoir (of infection) as an ecologic system in which an infectious agent survives indefinitely. Such a system includes all the component host populations, including that of any intermediate host or vector, in the context of any environmental component, and any quantitative requisite such as critical community size, which is required to maintain the agent indefinitely.

Vertebrate hosts that form an essential part of the system are reservoir hosts, though whether a whale or a fish is the reservoir host of *Anisakis* species can be a matter of debate. A host that becomes infected, but is not required for the maintenance of the population of a pathogen, can usefully be called an incidental host. (Accidental host is frequently used, but this is arguably a teleological term and therefore undesirable.) For incidental hosts that transmit pathogens from a reservoir to another incidental host, analogous to a pipe leading from a water reservoir, Garnham (*4*) coined the useful term “liaison host.”

Haydon et al. dismiss my definition on two grounds. First, I exclude liaison hosts from the reservoir. This distinction is valid and could be argued either way, but I suggest that the pipes leading from a reservoir do not form part of the reservoir and that it is both conceptually and practically important to distinguish liaison hosts from reservoir hosts. The second objection is that many pathogens depend on the presence of several host species, at any given stage in the life history, for their maintenance. This concept is clearly considered in my article: together, such hosts collectively constitute part of the reservoir system, though no single one may be the reservoir host in its own right.

In good scientific English, each term should have a precise definition, and synonyms should be avoided. The Oxford English Dictionary (OED) (*5*) definitions of reservoir generally refer to a place or container used for the collection and storage of water, other fluids, or even solid material.

The OED definition of reservoir as a medical term is. “A population which is chronically infested with the causative agent of a disease and can infect other populations.” While one might argue with the terms chronically, infested, the infection of populations, this definition captures the usual sense in which reservoir host is used.

The quotations given in OED are more helpful. The earliest one given for reservoir in a medical context is from 1937, “For the continuous existence of a disease there must be some reservoir of infection…” The most important reservoirs of infection are human or animal cases or carriers. Plants may be the reservoir of infection in some of the mycoses. However, according to OED, the compound term “reservoir host” was used earlier, in 1913, “The monkey is most probably the normal reservoir host [for *Physaloptera mordens*].”

The main conceptual difference between the proposal of Haydon et al. and my own is that mine is more generalized: for a given pathogen in a given place, there is a single reservoir. The proposal of Haydon et al. is more limited to practical considerations: the reservoir for one target host may not be the same as that for another target host in the same place.

The most important contribution of these two publications is that they raise an issue that has confused the literature for many years. Parasitologists, virologists, and bacteriologists should agree on a consensus set of terms for the ecologic description of multihost systems. When we all agree on what we are talking about, we will understand each other better.
